# Emergency Department Care Coordination Program for Assisted Living Residents With Dementia: A Qualitative Study

**DOI:** 10.1001/jamanetworkopen.2025.26413

**Published:** 2025-08-11

**Authors:** Grace F. Wittenberg, Peter T. Serina, Nichole E. Stetten, Ann Reddy, Ellen McCreedy

**Affiliations:** 1 Center for Long-Term Care Quality & Innovation, Brown University School of Public Health, Providence, Rhode Island; 2 Center for Gerontology & Healthcare Research, Brown University School of Public Health, Providence, Rhode Island; 3 Department of Emergency Medicine, The Warren Alpert Medical School of Brown University, Providence, Rhode Island; 4 Emergency Department, Massachusetts General Brigham Salem Hospital, Salem, Massachusetts; 5 Survey Research Center, Brown University School of Public Health, Providence, Rhode Island; 6 Department of Health Services, Policy & Practice, Brown University School of Public Health, Providence, Rhode Island

## Abstract

**Importance:**

Care transitions to the emergency department (ED) from assisted living centers (ALCs) for residents may include incomplete or inaccurate information during transfer. These transitions can be especially difficult for vulnerable populations, including persons living with dementia (PLWD).

**Objective:**

To assess perceptions of complex care managers (CCMs) implementing a care coordination program designed to improve communication for transfers from ALCs to the ED.

**Design, Setting, and Participants:**

This qualitative study analyzed semistructured video conference interviews with CCMs in February 2024. The ED early response program was available through a physician services group (Bluestone Physician Services), which provides care to residents in ALCs in Florida, Minnesota, and Wisconsin for patients in its accountable care organization. The physician services group identified the CCMs based on scheduling convenience. Data were analyzed in March and April 2024 using directed content analysis.

**Exposure:**

The ED early response program included electronic notification to the physician services group CCMs when a patient registered at an ED. CCMs then communicated via fax and telephone with the ED staff to provide key clinical information.

**Main Outcomes and Measures:**

CCM-perceived strengths and weaknesses of the program.

**Results:**

Of 22 total CCMs, 12 participated in this study (employed as a CCM for a median [IQR] of 2 [1-3] years; 12 [100%] female) and identified populations that they perceived to especially benefit from the program, including PLWD, patients in hospice, and patients living in group homes. CCMs shared how they communicated with various ED staff, including nurses and physicians, and that receptivity varied among staff. Strengths of the program include CCM advocacy for patients and program adaptability. CCMs described areas of opportunity as lack of education about the program among ED staff and lack of 24-hour coverage for CCMs. Overall, the perception shared by the CCMs was that the program positively affected both the ED experience for patients and the facilitation of goal-concordant care.

**Conclusions and Relevance:**

In this qualitative study of a care coordination intervention, CCMs advocated for their patients remotely by filling information gaps, particularly for PLWD and patients in hospice, and perceived that the intervention was associated with improved patient care. CCMs also identified key areas for improvement, such as to increase ED staff awareness of the program and to expand program hours. This care coordination intervention may provide an opportunity to address gaps in care for individuals living in ALCs who present to the ED.

## Key Points

**Question:**

Is a care coordination intervention led by complex care managers (CCMs) employed by an independent physician group associated with improved communication between the primary care team and emergency department (ED) staff for ED patients who reside in assisted living communities?

**Findings:**

In this qualitative study, semistructured interviews with 12 CCMs were conducted, and 5 overarching themes were identified. CCMs described their perceptions that the intervention improved advocacy for patients, especially those with dementia, and enhanced goal-concordant care, while also recognizing program opportunity areas, including improving ED staff education.

**Meaning:**

Interviews with the CCMs participating in this qualitative study indicated their positive impressions of the program, including perceived enhancement in care for patients.

## Teaser

This qualitative study assesses whether complex care managers leading a care coordination intervention perceive that it is associated with improved communication between the primary care team and emergency department staff for emergency department patients who reside in assisted living communities.

## Introduction

Over 800 000 older adults reside in assisted living communities (ALCs) in the US.^1^ ALC residents have increasingly higher rates of comorbidities and cognitive impairment and limited clinical staffing to address this increasing complexity.^2^ As ALC resident health care needs increase, unscheduled assistance, such as visits to the emergency department (ED), increase. A total of 49% of ALC residents visit the ED at least once each year.^3^ With each transition in care, there is an increased risk of duplicative testing, delays in care, and avoidable hospitalizations due to incomplete or inaccurate information during transfer.^4^ Residents of ALCs experience higher rates of ED utilization, longer stays in the ED, and higher resource utilization within the ED than older adults not living in long-term care facilities.^5,6^

Care transitions are particularly fraught for vulnerable patient populations, especially for persons living with dementia (PLWD).^7^ The traditional ED care model focuses on rapid evaluation and stabilization of acute conditions, but there is a gap in research to support optimal ED care practices for PLWD.^8^ Lack of patient information on transfer substantially impacts the care of PLWD, who cannot always provide an accurate, succinct medical history for ED health care professionals. often leading to even more testing, increased length of stay, and hospitalizations.^9,10^ Increased ED and hospital utilization is associated with adverse outcomes for PLWD, including delirium, falls, and accelerated cognitive and functional decline^11,12,13^ as well as increased return visits and mortality if discharged from the ED.^14,15,16^

To address information gaps during ALC to ED transitions, a physician services group (Bluestone Physician Services), developed a care coordination program (the ED Early Response Program). This physician services group serves more than 15 000 patients at approximately 575 ALCs and 475 group homes across 3 states (Florida, Minnesota, and Wisconsin). For patients within the entity’s accountable care organization (ACO), 70% of whom are PLWD, complex care managers (CCMs) support transitions of care by improving communication between hospitals, postacute settings, primary care professionals, families, and ALCs. CCMs are health care professionals, often nurses or social workers, providing comprehensive care coordination and support to ACO patients. In the ED early response program, CCMs are notified electronically when an ACO patient is registered at an ED and provide real-time, structured clinical information within 1 to 2 hours of patient ED registration. The goal of this qualitative study was to assess the strengths and weaknesses associated with the ED early response program, as perceived by the CCMs implementing the program.

## Methods

### ED Early Response Program

The physician services group developed an ED early response program, a standardized communication tool designed to relay key clinical information and outpatient resources available to the ED care team in real time (Table 1). When an ACO patient is registered in the ED, CCMs receive an electronic notification. Once notified, CCMs complete a medical record review of the existing visit and prior visits across various electronic medical records (EMRs), fax a standardized packet of patient information, and call the ED to relay clinical information (eAppendix 1 in Supplement 1).The Brown University Institutional Review Board in Providence, Rhode Island, approved this qualitative study, and it was determined to be exempt from informed consent because participants in the study were determined to be key informants with the focus on the ED early response program and the research posed minimal risk to participants. Study procedures adhered to the Consolidated Criteria for Reporting Qualitative Research (COREQ) reporting guideline in reporting our findings.^17^

**Table 1.** Procedural Steps of the ED Early Response Program

Step	Description
1	CCMs will be notified electronically when a patient registers at an ED through ADT message or email
2	CCMs will complete a medical record review within the physician services group EMR
3	CCM will fax the following information to the ED: Patient’s medications Power of attorney contact PCP and CCM contact information
4	CCM will call if during business hours (8:00 am to 4:00 pm) and relay the following clinical information and available outpatient resources: Any recent concerns or changes Significant medical history (eg, cancer, COPD, falls, etc that most impact ED assessment) Recent critical laboratory results or critical vital signs (especially low pulse oximetry reading, or low or high blood pressure) Baseline mental status (important to establish for ED) Any recent (within the month) ED visits or hospitalizations Code status Behavioral health provider suggestions Outpatient resources available through the physician services group
5	CCMs will document results of the interaction on the ED Early Response tracker sheet

Abbreviations: ADT, admission-discharge-transfer; CCM, complex case manager; COPD, chronic obstructive pulmonary disorder; ED, emergency department; EMR, electronic medical record; PCP, primary care provider.

### Study Design and Participants

The study spanned from November 2023 to June 2024, and the interviews took place in February 2024. We conducted 30-minute semistructured interviews with the CCMs who implemented the program. The physician services group identified 12 CCMs of 22 total based on scheduling convenience. Participants provided verbal consent to record, and interviews were held via video conferencing software (Zoom; Zoom Communications Inc). All sessions were recorded and transcribed verbatim via a paid voice and audio transcription (Rev Transcription Services). Participants were given a $25 gift card for participation.

### Participant Checking

After initial coding and analysis, themes and representative quotations were presented to CCMs (10 of 12 interviewed) and the national CCM manager in a focus group held via video conference for participant checking.^18,19^ All responses were audio recorded and reviewed by the study team (G.F.W., P.T.S., and N.E.S.). CCMs agreed with the current results reported herein. During the participant checking focus group, the CCMs added additional items not previously discussed.

### Data Analysis

A semistructured interview guide was developed (eAppendix 2 in Supplement 1) to assess the program.^20^ We used directed content analysis to synthesize data and develop themes from the interviews.^21^ We combined a deductive and inductive approach using general concepts from the structured interview guide to develop the initial codebook and added new codes as they emerged. Data were analyzed using the constant comparison method until saturation was reached (eTable in Supplement 1).^22^ All data were analyzed in NVivo software, version 14 (Lumivero).^23^ The reflexivity statement is located in eAppendix 3 in Supplement 1.

## Results

We interviewed a total of 12 CCMs (12 [100%] female). Participants worked in Minnesota (n = 7) and Florida (n = 5)]. The CCMs had a median (IQR) of 2 (1-3) years employed as a CCM and called an ED a median (IQR) of 91 (70-120) times from June 2023 to February 2024. A total of 5 themes were found: (1) population served (eg, PLWD, hospice patients); (2) program procedure; (3) program strengths; (4) areas of opportunity; and (5) associations with patient care (Table 2). Exemplar quotations for the themes are shared in the Box.

**Table 2.** Summary of Themes and Representative Quotations

Theme	Description	Exemplar quotation
Population served	Individuals or groups who are affected by the intervention	“I was able to explain how it [fall] happened and also what her [patient’s] baseline was, which was very important because she is nonverbal usually and she’s behavioral. And so knowing that information, they were able to feel comfortable sending her back [to her ALC] after scans looked fine.” [CCM 6]
Program procedure	Components of the intervention that are performed by CCMs from initial notification through documentation	“We have really gotten down the documentation to what we absolutely need and what is useful.” [CCM 12]
Notification & medical record review	Steps of the process from notification about patient registration in the ED to subsequent medical record review	“So I look into [hospital EMR], look at the ED nursing notes or provider notes, whatever’s available, to see what’s going on. And then looking through our [physician service] records, our provider notes, that helps me kind of gauge a snapshot of what’s been going on with the patient.” [CCM 2]
CCM communication	CCM conversations via phone and fax with the ED staff	“My job and my goal is to contact the hospital, give them all the information that they may need in order to properly care for that patient.” [CCM 1]
Physician services group documentation	Documentation of the results of the ED encounter using a tracking sheet	“Whenever we follow a patient, we have specific CCM documentation. It’s kind of a flow sheet.… I’ll document when each step happens.” [CCM 3]
Strengths	Aspects of the program positively associated with program implementation and patient outcomes	“I think it puts them more in the forefront of the nurse’s mind, knowing that somebody is caring about them and they’re worth it.” [CCM 12]
Program adaptation	The ability to change program procedures based on CCM feedback	“We played with a time frame, depending on should we call within the hour or maybe extend it to 1 to 2 hours and stuff like that. That, I feel like, has been helpful.” [CCM 9]
Positive receptivity	ED staff and systems being open to participate in the intervention	“I think generally most of the people I’ve talked to in the EDs have been appreciative.” [CCM 2]
Areas of opportunity	Aspects of the intervention that may be improved	“Sometimes we don’t get an ADT alert or it’s late, and that is really for a multitude of different reasons.” [CCM 5]
Education	How education or lack thereof appears to affect the program	“I do feel like maybe the [ED staff] aren’t educated enough about what our program does.” [CCM 10]
Lack of or neutral receptivity	ED staff and systems displaying a lack of openness or neutral receptivity to the program	“Some of the floor nurses, they’re super busy, they don’t have a whole lot of time to have a conversation with me, or if I do have them pass on information, sometimes it just gets forgotten about or things come up.” [CCM 1]
CCM working hours	How CCM working hours (8:00 am to 4:00 pm) may affect the program	“I think it would be nice if there were a way to maybe extend hours or do something like that to where we could even... Because a lot of the times, patients may fall or may have accidents at night when we’re not able to catch them.” [CCM 9]
Patient impact	How the program is broadly associated with patient care and experience	“[Families] feel a little safer with their loved one going into the [ED] knowing that we’re calling and they’re going to get that information.” [CCM 5]
Experience of ED visit	The association of the program with patient experience in the ED	“I feel like where the effectiveness is, is just driving maybe the duration of that ED visit, and getting straight to the point versus having to do numerous tests or trying to rule out other things.” [CCM 4]
Goal-concordant care	How the program is associated with aligning care and patient values and preferences	“I feel like [the ED Early Response Program is] pretty significant because for these specific patients, we keep it around what their wish is. So, what would they prefer.” [CCM 9]

Abbreviations: ADT, admission-discharge-transfer; ALC, assisted living center; CCM 1-6 and 9-12, complex case manager number; ED, emergency department.

Box. **Exemplar Quotations for Themes and Subthemes**

**Population Served**

“Let’s take, for instance, the hospice patient. I obviously want them to turn around and go back to the community. We don’t really want them to stay inpatient, because that’s not the purpose of hospice unless you absolutely need it.” [complex care manager (CCM) 7]
“A lot of emergency rooms get a confused patient. They don’t really have any information. There’s no family there, and they’re like, ‘Does this person normally not speak?’ What’s their orientation? We have no idea. And where are they from? They think they live in Texas. So us just calling, I think it’s helpful for me to say they’re from this assisted living memory care. This is the number to call for nursing. This is their family member, X, Y, and Z. Just so they get that baseline information. They normally are in a wheelchair. This is normal for them not to be ambulating, things like that. So I think that’s helpful.” [CCM 6]
“I don’t think there is such a thing as overcommunication and the fact that our primary care doctor notes don’t show up in the hospital’s electronic medical records, it’s like they’re starting at ground zero for every patient.” [CCM 8]

**Program Procedure**

**Notification and Medical Record Review**

“So we have our bridge, which everyone has a demographics sheet. So that is our main portal of communication, it has their code status, their insurance, their drug allergies, what group home they’re at. So I conduct a chart review. I review last primary care visit notes, if there’s a POA [power of attorney] or health care directive on file if there’s a SLUMS [Saint Louis University Mental Status Examination], recent transitions that they’ve had. If they’ve been in the Epic system, I’ll review if they’ve had recent visits, just kind of anything pertaining to what would be contributing to that visit that day.” [CCM 4]

**CCM Communication**

“We’re able to give more information, more in-depth information. Like, ‘Listen, this is what happened. This is what we’re doing on our end. This is what we’re seeing. This is not how the patient usually acts.’” [CCM 9]
“And then I would say anything else that I can gather that’s relevant. If they’re in for a mental health diagnosis and they see one of our psych providers, I might send some of those notes, or anything that seems relevant, once I know what’s going on.” [CCM 7]

**Physician Services Group Documentation**

“We chart for our work all the time, whether it’s an ED [emergency department] visit or just our normal everyday work, it’s in the same note flow... [and] for the [ED] Early Response Program… there is a specific part of the template.” [CCM 8]

**Strengths**

“I think you have somebody in your corner advocating for your goals of care and providing additional insight. I think it’s always a positive.” [CCM 11]

**Program Adaptation**

“We’ve pivoted from calling right away to calling at least within 2 hours just to allow time for triage to take place, which I’ve found helpful waiting. There’s a little more value added when they’ve seen the patient.” [CCM 11]
“Well, as far as clinical judgment, and we have a lot of meetings about this and in different situations, what would you do, what’s the standard, the practice standard, and it’s fine to make a standard, but then there are always exceptions…. And we have the freedom and the ability to use our clinical judgment based on each situation, not making it so cookie cutter.” [CCM 12]

**Positive Receptivity**

“I think [the ED care team is] very appreciative. They often express gratitude for the information. You’re filling in all of the blanks of what’s even happening.” [CCM 11]
“But I feel like maybe the receptiveness is due to how many people call that hospital, because there’s a lot of us care managers that call the same hospitals. So they feel comfortable with us.” [CCM 2]

**Areas of Opportunity**

“If we could improve our education and our relationships specifically with these [EDs], I think they would be more receptive in listening to us. I think we have more success with that. It’s like right time, right focus. And when it all hits, it’s perfect, but it also can go the other way.” [CCM 3]

**Education**

“I remember I had one nurse say to me, ‘I’ve never had a patient’s doctor call here before.’ They’re thinking old school thinking like the brick and mortar clinic where the person is seen once a year. And so I feel like, not so much anymore, but I feel like in the beginning, it was a lot of explaining what [the physician services group] does. And a lot of having to say that we go out, our providers, our teams are very involved in the patient’s care, and they see them on a regular basis. This isn’t the once a month physical at the clinic that you’re going to. These are providers that are in their home seeing them involved in every little bit, on call 24 hours a day via the Bridge and can be asked a question or follow-up at any time.” [CCM 5]

**Lack of or Neutral Receptivity**

“I feel like, I guess it just depends on what’s going on in the ED. If they’re super busy, maybe that could be the reason why they’re not so receptive.” [CCM 5]
“But it just came up in a meeting last week that a lot of people are getting pushback.… Some providers in the ED feel like, oh, here’s [Bluestone] trying to tell us what to do.” [CCM 5]

**CCM Working Hours**

“If I think of it on the side of the patient, what I would do to make it better is have it be a 24 hour, 7 days a week program, not just during business hours. And that’s a huge undertaking and a huge ask. I think it really could avoid a lot more inpatient hospitalizations. It could avoid a lot more unnecessary testing.” [CCM 12]

**Patient Impact**

**Experience of ED Visit**

“Sometimes I’ve had doctors be like, ‘I had no idea they had that diagnosis. I had no idea.’” So we don’t have to run all of these tests; we can just run these 2 because we already know this information now.” [CCM 7]
“I had a recent patient who had a couple of falls. She had already fractured her wrist at her assisted living. She went into the ED, I think, for another fall. So I contacted them and let them know she’s already been following up with ortho, she’s already open to PT, OT at her assisted living. And so they were able to send her back. They checked her out and made sure she was okay, but they felt comfortable sending her back to her assisted living because she already had these services in place.” [CCM 2]

**Goal-Concordant Care**

“I think it’s important for [the ED health care professionals] to know if they are always confused times 4 at a baseline and nonambulatory that they know who their decision maker is right away, that they’re doing things aligned with patients’ goals of care.” [CCM 11]
“One certain case that was really big for me is that one of our patients got sent in and they happened to be on hospice and family was like, ‘No, we’re not sending them to the hospital.’ That’s not the hospice philosophy. She was sent in—I don’t even remember why—but I made sure to call the hospital right away. They didn’t even know that she was on hospice and that family didn’t want her there. It was good to have that communication so they knew what her goals of care were and that they could just send her right back and not even have to start testing or start labs or do any of that stuff.” [CCM 1]

### Population Served

The CCMs identified patients with cognitive impairment and mental health conditions, patients in hospice, and patients living in group homes as key populations served by this program. Most CCMs considered it vital to ensure the ED workup and subsequent hospitalization were within a patient’s goals of care, particularly those in hospice.

With the assurance of close follow-up and a clear history provided by the CCMs, some patients were sent home after an appropriate ED evaluation. Examples included patients who already had an extensive outpatient workup, patients with dehydration, and patients who fell but were deemed safe for discharge. Conversely, even with the program in place, specific patients still required hospitalization, including those with severe pneumonia, stroke, or suicidality. The CCMs perceived information relayed through the program as being particularly impactful for patients who could not advocate for themselves.

There was disagreement among the CCMs regarding whether there was a population that did not benefit from the program. One CCM noted that the program may not be as helpful for patients who had “100% no cognitive deficit [and] able to tell their whole story” and another CCM noted that for simple finite issues, all the extra information provided by CCMs may not be pertinent.

### Program Procedure

The CCMs described the program procedure to be notification and medical record review, CCM communication, and documentation (Table 1). The CCMs felt that their knowledge of their patients, the health system, and how the ED functioned allowed for real-time adaptations to ensure key information was available to ED staff.

#### Notification and Medical Record Review

The CCMs covered their assigned patient panel and sometimes covered for colleagues. The CCMs were familiar with the patient, making the medical record review easier. When one of their patients registered at the ED, CCMs received notification via email or an admission-discharge-transfer notification. Several CCMs noted that on occasion, ALC staff would inform them of ED transfers via a secure communication hub. Most CCMs emphasized the breadth of patient information and sources available to them to review before calling ED staff for a given patient, including through the physician services group EMR and hospital EMR.

#### CCM Communication

The CCM communication with ED staff, families, and patients was intertwined with all aspects of the program, not only as a part of the process but also how it affected patients and the identified strengths and areas of opportunity. Most CCMs mentioned that the process began with sending a concise, standardized fax packet of patient information, followed by a phone call to the ED within 2 hours, to provide the patient’s history in addition to information that may or may not have been available through the EMR of each ED.

The CCMs often connected with ED nurses; however, communication also included contacting the physician, advanced practice provider, hospital case managers and social workers, the physician services group primary care team, the hospice team (when applicable), and families. The CCMs contacted ALCs for additional information as necessary. The CCMs highlighted functional and cognitive baselines, code status, family contacts, pertinent events, and recent medical history leading up to the ED visit. Despite the process being standardized, several CCMs mentioned the importance of adapting information to the specific patient and ED visit.

#### Physician Services Group Documentation

The CCMs had clear documentation procedures to support transitions of care. The CCMs documented information in multiple parts of the physician services group EMR, including the medical record, the secure communication portal, and an internal tracking flow sheet for monitoring process measures such as the time of ED registration, time of the CCM phone call, reason for transfer, ED staff contacted, and patient disposition.

### Strengths

The CCMs highlighted multiple strengths of the program, specifically citing their own contributions as key to its success. Half of those interviewed described themselves as a support or advocate for their patients. In addition to supporting patients, the CCMs cited the support they provided to ED staff, hospital staff, families, and primary care team with their clinical experience and understanding of the health system. They felt that they could leverage these relationships to ensure the care team had a shared understanding of each patient’s health status and goals.

#### Program Adaptation

The CCMs highlighted the major strength of the program as its adaptability. Examples cited by the CCMs included streamlining the fax packet sent to the ED, removing unnecessary documentation steps, and having a centralized spreadsheet with up-to-date phone and fax numbers for each ED. Most CCMs highlighted the change since the inception of the program from requiring contact with the ED staff within 1 hour of registration to an approximate 2-hour response time based on CCM feedback. The CCMs also highlighted the need and ability for adaptability in real time, having the flexibility to change depending on the clinical situation and needs of the patient.

#### Positive Receptivity

Most CCMs described a sense of positive receptivity on the part of ED staff as a strength of the program. The CCMs believed that once ED staff understood the aims of the program, they saw the program as highly effective and beneficial for patients. The CCMs also identified factors associated with increased receptivity, including familiarity with the program and with the physician services group. They also noticed increased receptivity when the ED staff were not overly busy.

### Areas of Opportunity

Key areas of opportunity included education, lack of or neutral receptivity, and CCM working hours.

#### Education

The subtheme of education and the lack of receptivity were often intertwined, with a lack of receptivity attributed to lack of education among ED staff about the program. The CCMs noted a lack of education about program goals and how CCMs could be a resource to ED staff. The majority of CCMs felt that if ED staff could be educated on the purpose of the program and how it could improve patient care, they would be more receptive. The CCMs proposed ideas on how to improve responsiveness, including offering pamphlets, conducting on-site visits by CCMs to the EDs with larger numbers of ACO patient visits, and connecting with ED leadership.

#### Lack of or Neutral Receptivity

The CCMs found variable receptivity depending on the hospital and the ED staff. The CCMs identified a lack of understanding of the benefits of the program and an incredibly busy work environment as reasons for neutral receptivity. There was also some concern, despite efforts by the CCMs to only provide accurate history, that some ED staff felt that CCMs were trying to direct care.

#### CCM Working Hours

Most CCMs also highlighted the program hours, weekdays between 9:00 am and 5:00 pm, as a limitation, given that many visits to the ED happened after regular working hours. Finding coverage for these times and the funding for continued use of the program was a barrier.

### Perceived Patient Impact

All CCMs perceived the ED early response program to be positively associated with patient care.

#### Experience of ED Visit

All CCMs felt that the information provided through ED early response program affected the experience of the ED visit. By providing context, they felt that they were able to reduce diagnostic testing, unnecessary admissions, and the time the patient spent in the ED. This context included the patient’s known comorbidities and an updated medication list, the specific events leading to the ED visit, outpatient tests or imaging, or resources available to the patient outside the hospital already in place. One CCM gave an example of a patient already with the necessary specialty follow-up who was able to go home after her trauma workup for a fall.

#### Goal-Concordant Care

The majority of the CCMs also highlighted how they felt that the program helped ensure ED care aligned with each patient’s goals of care, particularly for the PLWD. The CCMs involved family members or other decision-makers in the conversations about goals of care and saw themselves as advocates for the PLWD. The CCMs also identified patients in hospice as individuals who benefited from goal-concordant care. The majority of CCMs shared that providing information to the ED staff about the hospice patient’s status and goals of care prevented unnecessary hospitalizations and allowed patients in hospice to return home expeditiously.

### Participant Checking

Overall, the CCMs felt that the themes identified by the research team summarized their views of the ED early response program accurately and appropriately. Each theme was supported by additional supporting information to the theme rather than new content. For example, for *population served* (theme 1), the CCMs emphasized the importance of the program for patients who could not advocate for themselves, including patients living in group homes. For *program procedure* (theme 2), the CCMs again emphasized the importance of their documentation not only for the program but also as a part of their work for patients across the continuum of care. When discussing *strengths* (theme 3), the CCMs reiterated the importance of their knowledge of patients prior to the ED visit and that changing CCM response time to 2 hours assisted with additional medical record review. For *areas of opportunity* (theme 4), they again discussed how busy staff at EDs seemed to be and the importance of balancing the need to share key information while being respectful of ED staff time. For *patient impact* (theme 5), many CCMs verbally agreed that the program was positively associated with patient care and patient experience in the ED.

## Discussion

Overall, this qualitative analysis of interviews with CCMs identified populations, including PLWD and individuals in hospice, associated with the greatest benefits from the ED early response program. The CCMs described their communication with ED staff, families, and other care team members as essential to the program procedure. A key program strength was the ability of the CCMs to adapt the program to each ED interaction. A lack of education regarding the existence and purpose of the program was a key opportunity for improvement. The CCMs felt that some ED staff found the program useful, while others were less receptive to a call from the CCMs. The CCMs shared that the program enabled them to provide additional context to enhance the ED visit, through reduction in diagnostic testing and unnecessary admissions, and to better align with each patient’s goals of care, especially PLWD.

There has been notable research^9,24^ on ED to community transitions of care and subsequent patient outcomes for residents in nursing homes, but there is less literature describing ALC to ED transitions. Although some of the findings may be extrapolated to care transitions broadly, there are few programs, such as the ED early response program, that are designed specifically to improve ALC to ED care transitions. ED transfer forms and transfer checklists have been described in the existing literature as enhancing the consistency of information shared between nursing homes and EDs.^25^ Unlike checklists, a strength of ED Early Response Program included increased adaptability for implementation in the clinical setting and direct communication between a CCM and ED staff. Despite a variety of care coordination interventions^26^ designed to alleviate transitions, barriers to effective transitions between nursing homes and EDs continue due to the complexity of older adults, who often have multiple comorbidities and may have cognitive impairment. Notably, barriers include staff burden (comfortability of caring for older adults with acute concerns), discontinuity of care (health care professionals lacking patient information during transitions), and transitions negatively impacting resident well-being.^27^ The ED early response program addresses these barriers by improving communication, which may alleviate staff burden and lead to decreased diagnostic testing and hospitalizations and may enhance goal-concordant care, specifically for PLWD in ALCs.

The ED early response program was received positively by the CCMs administering it. The strength of the program lies in the expertise and dedicated time to facilitating care transitions. CCMs have access to key clinical information and can facilitate real-time interprofessional communication between the ALCs, primary care providers, families, specialists, and ED staff. By ensuring accurate, up-to-date information on each patient’s functional and cognitive baseline, medical history, and events preceding the ED visit, the program may reduce redundant testing and unnecessary hospitalizations and align patient care with their specific goals. This is particularly impactful for PLWD, who often struggle with communicating their medical histories and care preferences. There are unique considerations for PLWD when presenting to the ED, including a lack of familiarity with the environment and increased care partner burden to share information with health care professionals about their loved one.^28^ The ED early response program seeks to alleviate these burdens for PLWD and their caregivers through direct communication with the ED team, ultimately providing additional opportunity to recenter on each individual’s goals of care.

### Limitations

This study has several limitations. First, interviews were restricted to 30 minutes due to busy schedules and the limited availability of CCMs. The physician services group leadership also chose the CCMs based on availability, and we were unable to interview any CCMs in Wisconsin, 1 of 3 markets the program services, which may have introduced bias into the selection process. Finally, we do not have ED staff or patient perspectives as key voices in determining the success of this program.

## Conclusions

The results of this qualitative analysis indicated that CCMs perceived the ED early response program as acceptable, appropriate, and feasible. The CCMs saw the innovation of this program as improving ED care for their patients by improving communication between primary care providers and the ED with a specific goal of reducing unnecessary hospitalizations. Future research should address ED health care professional, patient, and caregiver perspectives of the program.

## Notes

Supplement 1. **eAppendix 1.** Bluestone Fax Packet Sent to ED During ED Early Response Intervention **eAppendix 2.** In-Depth Interview Guide Bluestone Complex Care Managers (CCMs) **eAppendix 3.** Reflexivity Statement **eTable.** Codebook Including Codes, Subcodes, Explanations and Number of Times Each Code Is Referenced During CCM interviews

Supplement 2.
Data Sharing Statement
