# Supplementary material for: Emergency Department Care Coordination Program for Assisted Living Residents With Dementia: A Qualitative Study
Source: JAMA Netw Open. 2025 Aug 11;8(8):e2526413. doi: 10.1001/jamanetworkopen.2025.26413 (PMC12340648; doi:10.1001/jamanetworkopen.2025.26413)
Supplement: Supplement 1. — eAppendix 1. Bluestone Fax Packet Sent to ED During ED Early Response Intervention eAppendix 2. In-Depth Interview Guide Bluestone Complex Care Managers (CCMs) eAppendix 3. Reflexivity Statement eTable. Codebook Including Codes, Subcodes, Explanations and Number of Times Each Code Is Referenced During CCM interviews [file jamanetwopen-e2526413-s001.pdf]

Supplementary Online Content

Wittenberg GF, Serina PT, Stetten NE, Reddy A, McCreedy E, et al. Emergency department care coordination program for assisted living residents with dementia: a qualitative study. *JAMA Netw Open*. 2025;8(4):e223050. doi:10.1001/jamanetworkopen.2025.3050

eAppendix 1. Bluestone Fax Packet Sent to ED During ED Early Response Intervention

eAppendix 2. In-Depth Interview Guide Bluestone Complex Care Managers (CCMs)

eAppendix 3. Reflexivity Statement

eTable. Codebook Including Codes, Subcodes, Explanations and Number of Times Each Code Is Referenced During CCM interviews

This supplementary material has been provided by the authors to give readers additional information about their work.

© 2025 Wittenberg GF et al. *JAMA Network Open*.

eAppendix 1. Bluestone Fax Packet Sent to ED During ED Early Response Intervention

Bluestone Physician Services℠

270 North Main St. Suite 300 STILLWATER, MN 55082

FAX COVER SHEET

TO: Hospital/ emergency department name
FAX: Hospital/ emergency department fax #

RE: Patient Name

NOTES:
Please see attached documentation from patient's last primary care visit note. This patient is part of our ACO, and I am available to help with discharge planning needs when stabilized in your ED.

Our goal is to avoid hospitalization, this patient does NOT require a three night inpatient stay for direct admission to SNF/TCU (if medically indicated). I am available to assist you in any discharge needs from the ED.

Please reach out with any questions.

From: Complex Care Manager Name, Complex Care Manager
Phone: Complex Care Manager phone #

Primary Care Physician
Provider Name
Provider Bluestone Address
Office: # fax: #
MAIN FAX NUMBER:
Confidential Notice: The documents accompanying this transmission may contain confidential health information that is legally protected by state and federal law. This information is intended only for the use of the individual or entity named above. If you are not the intended recipient, you are hereby notified that any disclosure, copying, distribution, or taking of any action in

© 2025 Wittenberg GF et al. *JAMA Network Open*.

reliance on or regarding the contents of this faxed information is strictly prohibited. If you have received this fax in error, please immediately notify the sender by telephone: 651-342-4275, or email: medrecords@bluestonemd.com to arrange for the return or destruction of the original documents.

PATIENT SNAPSHOT:
Pertinent patient specific information regarding baseline, past medical history, acute events, etc.

CARE MANAGEMENT FACESHEET

PATIENT INFORMATION

Name: DOB:
Phone: Facility:

Address:

Service Site:

INSURANCE INFORMATION:

CONTACTS

PHARMACY

© 2025 Wittenberg GF et al. *JAMA Network Open*.

Bluestone
Accountable Care Organization

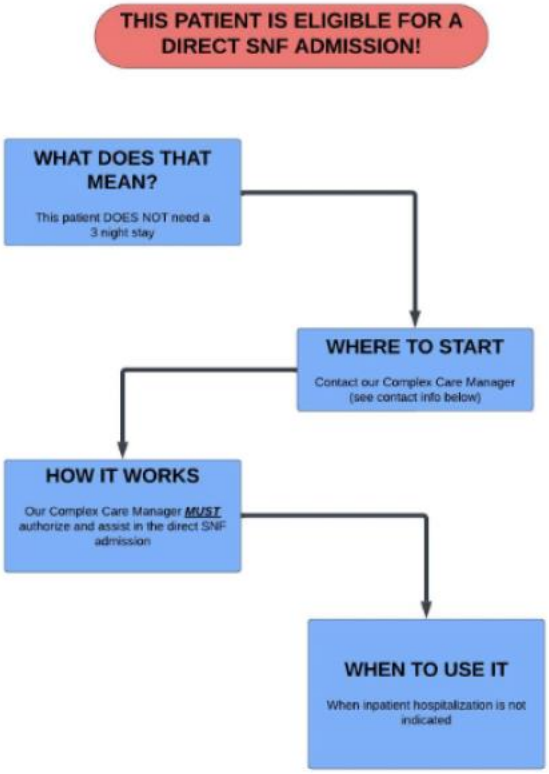

Care Manager Contact: Care Manager Contact information

LAST VISIT SUMMARY:
Last visit note completed by Bluestone PCP for this patient is inserted here. Includes details specific to visit, as well as:
History of Chronic Conditions, Social History, Past Medical History, Hospitalization History, Surgical History, Family History, Immunization History, Physical Exam, Allergies, Vitals Medications, Treatment plan

© 2025 Wittenberg GF et al. *JAMA Network Open*.

eAppendix 2. In-Depth Interview Guide Bluestone Complex Care Managers (CCMs)

Questions
<u>Background/Process</u>

Q1 Please describe your involvement with the ED Early Response program?

- Probe: In which state are you based?
- Probe: What are your responsibilities as a CCM?
- Probe: How many times have you participated in the ED Early Response program (i.e. how many times have you contacted the ED on the behalf of a Bluestone patient)

Q2 What is the process for communication between the ACM and the ED?

- Probe: How do you know there is a patient eligible for the ED Early Response Program? (Step 1)
  - How does this change if the ED visit occurs during off-hours (i.e. holidays, weekends, or 4p-8a)
- Probe: How do you obtain information about a patient that's in the ED? (Step 2)
- Probe: How do you contact the ED providers? (Step 3)
  - What if you are unable to contact the ED provider?
- Probe: What type of ED providers are you able to contact?
  - Is there variation in the response you get from different providers?
- Probe: What patient information do you provide to the ED provider?
  - What specific information do providers ask for ?
  - What information do providers not find helpful?
  - Are you able to obtain all the information providers find helpful?
- Probe: How do you fax information to the ED? (Step 4)
  - When you fax information to the ED, typically how many pages of information get faxed?
- Probe: Tell me how you document the results of the interaction. (Step 5)

<u>Impressions</u>
Q3 How do you think the ED Early Response program changes patient care, if at all?
- Probe: How does the information you provide by phone AND fax change tests performed by the ED, if at all?
- Probe: Does the information shared ever change the decision regarding admission to the hospital?
- Probe: Tell me any changes you have seen regarding the patient's disposition as a result of the information shared.
- Probe: Give me an example of a time when the information you provided was helpful.
- Probe: How about an example of when the information, or some of the information, did not seem to help…

<u>Health Equity</u>
Q4 What type of patient does this program help most?
- Probe: What type of patient does this progam help least?
- Probe: We are also looking for how to improve care for our highest need patients, how do you think this program affects these patients in particular?
  - Examples underserved populations: e.g. complex geriatric, chronic mental health, cognitive impairment, functional limitations, racial minorities, economically disadvantaged

<u>Strengths and Weaknesses</u>
Q5 What do you believe are some strengths of the ED Early Response program?
- Probe: Ask Ppt to provide further details on each strength identified here and earlier in the interview
- Probe: What else seems to be working well?
- Probe: What parts of the program should we absolutely keep?

Q6 What do you think would make the program better?
- Probe: Get suggestions for each challenge identified here and earlier in the interview
- Probe: What else needs to be improved?

© 2025 Wittenberg GF et al. *JAMA Network Open*.

- Probe: What parts should we get rid of?
- Probe: Do other CCMs that you have spoken with feel similarly? Or have any other suggestions?

Closing
Q7 Is there anything else you would like to add that we haven't already discussed?

© 2025 Wittenberg GF et al. *JAMA Network Open*.

eAppendix 3. Reflexivity Statement

The interview and analysis team included PS (a practicing emergency physician who received post-doctoral training in gerontology and qualitative methods), NS (a public health PhD researcher who is a qualitative methods expert), and GW (a trained research assistant and medical student with prior qualitative research experience). PS has a relationship with Bluestone leadership in the context of implementing this study and a previous community research project with Bluestone as a partner. GW served as an RA collecting qualitative data from a prior project with Bluestone. NS had no prior experience working with Bluestone and worked from a purely neutral standpoint and ensured rigorous qualitative methodology throughout the project. Although PS has a background as an emergency physician and a relationship with Bluestone with the potential to bring bias into the study and analysis, the other researchers' backgrounds and relationships help reduce this possible bias.

© 2025 Wittenberg GF et al. *JAMA Network Open*.

eTable. Codebook Including Codes, Subcodes, Explanations and Number of Times Each Code Is Referenced During CCM interviews

Numeric Nomenclature for Codes		Node Label	Explanation, Examples	Number of Reference s
**1**		**Intervention Procedure**	Intervention steps: This code is to help elucidate what specific components of the intervention done by CCMs. This process subgroups will likely get double coded with strengths/weakness/equity depending on comments. If the CCM discusses a procedural step, this is coded.	3
	1.1	Notification & Chart Review	Step 1 and step 2 in interview guide	35
	1.2	CCM Communication	Step 3 and step 4 in interview guide. Phone calls & Fax to exchange of information from CCMs to key stakeholders (e.g. ALF staff, ED staff, Bluestone providers, patients, families, etc). This is procedural step and the substance of the communication between CCMs and stakeholders	143
	1.3	Bluestone Documentation	Step 5 in interview guide	16
**2**		**Patient Impact**	Impact on patient care and experience. Note: if the quote doesn't clearly delineate between experience and goal concordant care, use the major theme of patient impact.	40
	2.1	Experience of ED visit	Impact of patient experience when the patient is within the ED (e.g. testing done, ED length of stay, avoidance of hospitalization, close follow up arranged, etc).	34
	2.2	Goal Concordant Care	Goal concordant care is achieved, or patient care that is the alignment with patient values and preferences.	15
**3**		**Strengths**	Strengths of the program or characteristics that make a positive impact on program implementation and patient outcomes. Code as general code, unless specific subcode is identified	54
	3.1	Program Adaptation	Ability to change the program procedures based on CCM feedback/active quality improvement; ability for CCMs to adapt based on specific patient needs	32

© 2025 Wittenberg GF et al. *JAMA Network Open*.

Numeric Nomenclature for Codes		Node Label	Explanation, Examples	Number of References
	3.2	Positive Receptivity	Openness to participation in the intervention and take action based on the information received	15
**4**		**Areas of opportunity**	Things missing or could be improved upon in the intervention. Code as general code, unless specific subcode is identified	33
	4.1	Education	Stakeholders not aware of the ED Early Response program or how it can help Bluestone patients presenting to the ED	16
	4.2	ACM working hours	ED Early Response only available office hours during week days	28
	4.3	Lack of/Neutral Receptivity	Lack of/neutral openness to participation in the intervention and take action based on the information received	32
**5**		**Population served**	Specific patient population for whom this intervention is most effective and beneficial	84

© 2025 Wittenberg GF et al. *JAMA Network Open*.
