# Supplementary material for: Emergency Department Care Coordination Program for Assisted Living Residents With Dementia: A Qualitative Study
Source: JAMA Netw Open. 2025 Aug 11;8(8):e2526413. doi: 10.1001/jamanetworkopen.2025.26413 (PMC12340648; doi:10.1001/jamanetworkopen.2025.26413)
Supplement: Supplement 2. — Data Sharing Statement [file jamanetwopen-e2526413-s002.pdf]

Data Sharing Statement

Wittenberg. Emergency Department Care Coordination Program for Assisted Living Residents With Dementia. *JAMA Netw Open*. Published August 11, 2025.
doi:10.1001/jamanetworkopen.2025.26413

Data
Data available: No
